# Frequency of uterine sarcomas in patients admitted for uterine fibroid surgery

**DOI:** 10.4274/jtgga.2016.0248

**Published:** 2017-06-01

**Authors:** Liselotte Mettler, Nicolai Maass, Khulkar Abdusattarova, Astrid Dempfle, Ibrahim Alkatout

**Affiliations:** 1 Department of Gynecology and Obstetrics, University Hospitals Schleswig-Holstein, Campus Kiel, Germany; 2 Institute of Medical Informatics and Statistics, University Hospital Schleswig-Holstein, Christian Albrecht-University, Kiel, Germany

**Keywords:** uterine fibroids, myomectomy, hysterectomy, uterine sarcoma

## Abstract

**Objective:**

To estimate the frequency of unsuspected uterine sarcoma identified postoperatively in women undergoing surgery for presumed benign uterine fibroids at a single university hospital.

**Material and Methods:**

This was a retrospective single-center study; the records of all 2275 patients with uterine fibroids and uterine sarcomas from 2003 to 2015 were reviewed. Descriptive statistics were used to analyze demographic and clinical characteristics. To calculate confidence intervals (CIs), the Clopper-Pearson Exact method was applied.

**Results:**

Preoperatively, 2269 patients had presumed benign uterine fibroids, and six patients had suspected uterine sarcoma. Among the 2269 patients who underwent surgery for presumed uterine fibroids, endometrial stromal sarcoma was histopathologically revealed in only one patient [0.044%, 95% CI: (0.001-0.25)] after laparoscopic subtotal hysterectomy with morcellation. All six patients who were preoperatively diagnosed having uterine sarcoma underwent direct conventional cancer treatment. Histopathologic analyses confirmed four cases of uterine leiomyosarcoma, one high-grade undifferentiated uterine sarcoma, and one embryonal rhabdomyosarcoma. Altogether, seven women were diagnosed as having uterine sarcomas over this twelve-year period.

**Conclusion:**

In our institution, the frequency of unsuspected uterine sarcomas was 1/2269 (0.044%) among women who underwent myomectomies and hysterectomies to treat presumed benign uterine fibroids.

## INTRODUCTION

The prevalence of unsuspected uterine sarcomas in those undergoing uterine fibroid surgery is of concern, and this issue is particularly important when laparoscopic power morcellation is used (1). Unintentional morcellation of uterine sarcomas can occur if a patient is misdiagnosed as having uterine fibroids or when a uterine sarcoma is found within a "fibroid" uterus. Preoperative diagnoses of uterine sarcomas are still difficult despite the implementation of various diagnostic methods, including biochemical and immunologic markers, imaging modalities, and ultrasound-guided core needle biopsies (2, 3, 4, 5). Diagnostic challenges can delay the accurate diagnosis of uterine sarcoma and may lead to inappropriate surgical procedures such as morcellation. The inadvertent morcellation of an undiagnosed uterine sarcoma can cause upstaging of a cancer and worsen the prognosis of the patient (6, 7).

Another potential problem is that a concern over the potential presence of sarcoma may decrease the frequency of laparoscopic surgeries to patients with uterine fibroids due to the risk associated with uterine sarcomas, which may negatively influence clinical outcomes. Minimally invasive surgery is offered to many patients with uterine fibroids because of its advantages over open surgery, which include less intraoperative blood loss, a reduced chance of postoperative wound infection, less post-operative pain, and shorter convalescence times (8). Thus, this study aimed to evaluate the frequency of postoperative histologic diagnoses of uterine sarcoma following myomectomy or hysterectomy for presumed benign uterine fibroids. Additionally, this study analyzed the clinical and immunohistopathologic characteristics of all uterine sarcoma cases during a twelve-year period in our clinic.

## MATERIAL AND METHODS

Data were collected retrospectively from the records of patients with uterine fibroids and uterine sarcomas treated between 2003 and 2015. The database included data from patients who signed an informed consent form allowing the use of their specimens and clinical data for research purposes. In compliance with ethical standards and local data protection regulations of the University Hospitals Schleswig-Holstein, anonymous data were generated for statistical analysis.

The files of patients who underwent hysterectomies and myomectomies for presumed benign uterine fibroids were reviewed according to the International Classification of Diseases code (D25) in combination with at least one German procedure classification code. Demographic and clinical data were only collected for women with uterine sarcomas and were not collected for women with only uterine fibroids. All cases of uterine sarcoma were assessed based on the morphologic codes of the International Classification of Diseases for Oncology, third edition (ICD-O-3). Uterine carcinosarcomas were not included in this study because they are no longer classified as a subtype of uterine sarcoma but instead are considered as uterine carcinomas. Unexpected sarcomas were defined as cases where uterine sarcoma was confirmed via postoperative pathologic analysis, in which it was not suspected preoperatively and there was no clinical preoperative suspicion or indication of malignancy. The staging of uterine sarcomas was performed based on the updated International Federation of Gynecology and Obstetrics Classification 2009. The following variables were retrieved from the medical records of the women who were diagnosed as having uterine sarcomas: patient age, body mass index (BMI), clinical symptoms, image modalities, intraoperative frozen-section examination results, surgical procedures performed, additional treatment, survival status, histologic evaluation and the results of immunohistochemical analyses, which included smooth muscle actin (SMA), CD 10, CD117, CEA, CA 19-9, SCC, Ki67, CA125, p53 vimentin, desmin, calponin, actin, h-caldesmon, estrogen receptors (ERs), progesterone receptors (PRs) and mitotic index measurements.

Statistical analyses were performed using the Statistical Package for the Social Sciences (SPSS, version 18.0). Data are presented as the mean and standard deviation for continuous variables and as percentages for categorical variables. To calculate the 95% confidence intervals (CIs) for the proportion of patients in each category based on the binomial distribution, the Clopper-Pearson Exact method was used.

## RESULTS

Between 2003 and 2015, 2269 women received a preoperative diagnosis of presumed benign uterine fibroids. Of these, 938 (41.3%) women had myomectomies and 1331 (58.7%) women had hysterectomies, only one patient was postoperatively diagnosed as having endometrial stromal sarcoma (ESS) based on a pathohistologic analysis; the patient was aged 48 years and her BMI was 26.5 (kg/m^2^) at the time of diagnosis, and she reported hyper and polymenorrhea. The patient’s medical history was otherwise unremarkable. Ultrasound examination showed a hypoechogenic lesion (8.5 cm) with a clear borderline on the left posterior wall of the uterus. Physical examination of the external genitalia, vagina and cervix showed no abnormalities. Pelvic examination revealed a uniformly enlarged uterus, and no adnexal masses were palpated. The primary diagnosis was symptomatic transmural leiomyoma. The patient underwent a laparoscopic subtotal hysterectomy (LSH) with morcellation. The final pathohistologic results detected ESS, and the specimen weighed 294 g. Immunohistochemical analysis showed the following results: CD10(+), desmin(+), SMA(−), actin(−) and 5% Ki67(+). Furthermore, immunohistochemical assays for ERs, PRs, and p53 showed the following results: ER (+++), PR (+++) and p53 (−). Two weeks after the initial surgery, a laparotomic removal of cervical stump with bilateral salpingo-oophorectomy and an omentectomy was performed. The final pathologic report described no signs of any metastatic lesions.

Six patients who were diagnosed preoperatively as having suspected uterine sarcoma were treated and followed up according to the treatment protocol for uterine sarcomas (see Table 1).

The four women diagnosed as having uterine leiomyosarcoma (ULMS) were postmenopausal, and their dominant presenting symptom was postmenopausal bleeding. The mean age of the women with ULMS at the time of diagnosis was 73.7±3.9 years (range, 68-79 years), and their mean BMI was 27.2±3.7 kg/m2 (range, 22.2-30.8 kg/m2). Two of these women had advanced-stage ULMS with multiple metastases at the time of diagnosis. Preoperative biopsy confirmed the suspicion of uterine sarcoma. Moreover, preoperative diagnostic testing resulted in a high clinical suspicion of uterine sarcoma due to the patients’ postmenopausal age, clinical factors, rapidly growing uterine mass, and the irregular appearance of the tumors detected via ultrasonographic examination, computerized tomography (CT) or magnetic resonance imaging (MRI) scans. Intraoperative “frozen section” analyses were performed, and the suspicion of malignancy was confirmed in each case. The results show that tumor cells were positive for calponin, SMA, and CD117, and that the proliferative activity of Ki-67 was elevated. Patients diagnosed as having ULMS were treated according to the treatment guidelines for ULMS, which include an exploratory laparotomy with radical hysterectomy and bilateral salpingo-oophorectomy. The mean uterus weight of women with ULMS was 873.5±410.8 g (range, 308-1128 g). In two patients, ULMS coexisted with leiomyoma. Definitive diagnoses of uterine sarcoma were obtained through histologic and immunohistochemical analyses. The immunohistochemical results were positive for SMA, CD 10, desmin, and h-caldesmon, and they indicated a high proliferative activity of Ki67. The mean mitotic index for the ULMS cases was 35.75±12.81 mitoses/HPF (range, 22-54).

A woman aged 48 years with high-grade undifferentiated uterine sarcoma (HGUS) presented to the emergency unit with acute abdominal pain and abnormal uterine bleeding. Her initial diagnosis was uterine fibroids. A hysteroscopy was performed to clarify the cause of bleeding. It was noted that the surface of the uterine fibroid observed via hysteroscopy was irregular, and the histologic analysis indicated HGUS. In addition to these results, a CT of the abdominal cavity revealed a 6x7-cm intramural tumor, no enlarged lymph nodes, and no evidence of abdominal metastases. Tumor markers CEA, CA 19-9, SCC were within normal ranges, but the CA125 level was elevated (230.5 units/mL). Consequently, an open hysterectomy with bilateral salpingo-oophorectomy and omentectomy was performed. The final pathologic report described no signs of any metastatic lesions. The weight of the uterus was 398 g, the size of the tumor was 5 cm, and the mitotic index was 12 mitoses/10 HPF.

A woman aged 67 years with embryonal rhabdomyosarcoma (ERMS) also had polycystic kidney disease and renal failure (hemodialysis since 2011), which resulted in secondary anemia and hyperparathyroidism. Her BMI was 34.6 kg/m2, and she had postmenopausal uterine bleeding. An endocervical curettage biopsy was performed, and the initial pathologic examination indicated HGUS. MRI revealed a heterogeneously enhanced huge mass, measuring approximately 15×10 cm in diameter, possibly arising from the uterine corpus and showing no definite evidence of metastases in the lymph nodes or other organs. A total laparoscopic hysterectomy with salpingo-oophorectomy was performed. The weight of the uterus was 300 g, and the size of the tumor was 5 cm. An immunohistochemical examination concluded that the ERMS cells were characteristically positive for vimentin and desmin, but were negative for CD10, calponin, and SMA. The frequency of unsuspected ULMS, HGUS, and ERMS was 0/2269. For ESS, it was 1/2269 [0.044%, 95% CI: (0.001-0.25)] among the women in this study who underwent myomectomies and hysterectomies for the treatment of presumed benign uterine fibroids. The total number of patients with uterine sarcoma was seven during the twelve-year period. Out of them, 6 were diagnosed through biopsy prior and during their larger surgical intervention, as described in Table 1. Only one ESS was diagnosed after a laparoscopic subtotal hysterectomy.

## DISCUSSION

Currently, there is no clear agreement among the available datasets on the prevalence of postoperative detection of uterine sarcoma associated with surgery for uterine fibroids. In our study, the frequency of unexpected ULMS in patients who underwent surgery for uterine fibroids was 0% (0/2269). Picerno et al. (9) presented a retrospective study that showed no cases of ULMS among 1004 women who underwent surgery for uterine fibroids. In addition, Pritts et al. (10) found a low percentage of these cases from a comprehensive analysis of 133 studies, in which there was a 0.051% prevalence of unsuspected ULMS among more than 30,000 women. A recent study from the Food and Drug Administration (FDA) that analyzed 12,402 women who underwent surgery for uterine fibroids estimated that the prevalence of unexpected ULMS was 0.064% (11). In a retrospective analysis of 8720 women who underwent laparoscopic supracervical hysterectomies for presumed uterine fibroids, Bojahr et al. (12) found that the postoperative histologic analyses revealed two cases of ULMS (0.023%). Recently, Kho et al. (13) conducted a prospective cohort study and found that among 10,119 women who underwent a hysterectomy for benign gynecologic indications, five unexpected cases of ULMS were identified, corresponding to a 0.049% incidence rate for unexpected ULMS.

The present study found that the frequency of unexpected ESS among women who underwent surgery for presumed benign uterine fibroids was 0.044% (1/2269). Other studies have reported the following statistics: Graebe et al. (14) identified three unexpected ESS cases among 1361 patients who underwent surgery for uterine fibroids (0.22%), Bojahr et al. (12) reported four unexpected cases of ESS among 10,119 laparoscopic supracervical hysterectomies (0.037%), and Kho et al. (13) reported two cases of unexpected ESS among 10,119 hysterectomies (0.019%).

Overall, our study found 6 cases of preoperatively suspected uterine sarcoma and one unsuspected case among 2269 patients undergoing myomectomy and hysterectomy who had indications of benign uterine fibroids during a 12-year period. Kho et al. (13) reported 64 cases of preoperatively suspected uterine sarcoma and 9 cases of unexpected uterine sarcoma among 10,119 hysterectomies performed due to benign indications within a 13-year period.

The prevalence of unexpected uterine sarcomas among patients undergoing uterine fibroid surgery appears to be low, but morcellation can negatively impact the patient’s future with regard to the recurrence of disease and survival. Bogani et al. (15) concluded that open power morcellation was associated with a 3- and 4-fold increase in overall and intra-abdominal recurrence of ULMS, respectively, as well as a 2.5-fold decrease in overall survival compared with patients whose tumors were removed intact. Guyon et al. (16) concluded that morcellation might expose patients to increased morbidity in cases of unrecognized malignancy due to the intra-abdominal dissemination of cancer.

Selecting the method of surgical treatment for patients with large uterine fibroids currently poses a dilemma for gynecologists due to the risks associated with myomectomy and morcellation in pre-malignant and malignant uterine tissue. Until a modified morcellation method, such as contained morcellation, can be agreed upon and implemented for clinical practice, it is important to consider the findings of a recent retrospective study. The study by Harris et al. (17) included a comparative analysis of 18,299 hysterectomies performed in the 15 months leading up to and the 8 months after the FDA safety communication was released in April 2014. The results show that the application of abdominal (1.7%) and vaginal hysterectomies (2.4%) increased, whereas there was a 4.1% decline in laparoscopic hysterectomies. An overall higher rate of complications was observed (excluding blood transfusions) from 2.2 to 2.8% after the date of the FDA safety communication, and the rate of hospital readmissions within 30 days also increased from 3.4 to 4.2% (17).

To decrease the risks of unintended morcellation of uterine sarcomas, a preoperative differential diagnosis between uterine fibroids and uterine sarcoma should be performed by utilizing a combination of clinical findings, image modalities, and immunologic and biochemical factors.

The main limitations of our study are its retrospective design and that it is a single-center study. Also, the results of post-operative screening for uterine cancers after myomectomy and cervical cancer screening after subtotal hysterectomy were not analyzed. The strength of this study is that the demographic data, detailed clinical data, and specific immunohistochemical markers were available for all cases of uterine sarcoma.

## CONCLUSION

The frequency of unsuspected uterine sarcoma was 1/2269 [0.044%, 95% CI: (0.001-0.25)] among the women in this study who underwent myomectomies and hysterectomies for the treatment of presumed benign uterine fibroids. The risk of uterine sarcoma after a preoperative selection of women with presumed benign fibroids appears to be very low.

## Figures and Tables

**Table 1:**
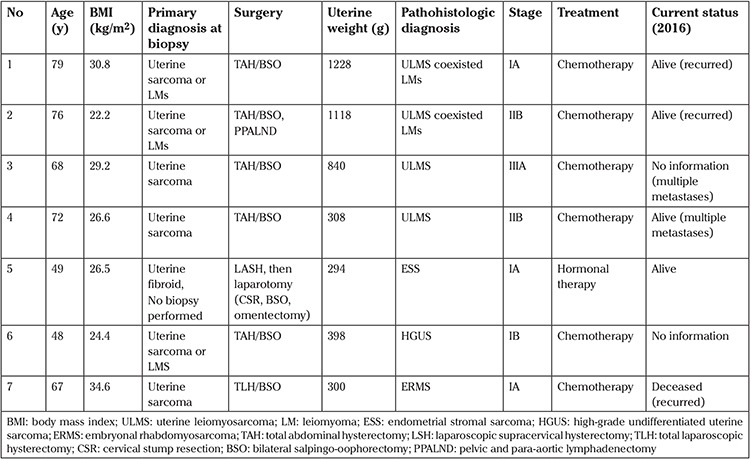
Cases of uterine sarcoma and endometrial stromal sarcoma in patients treated between 2003 and 2015 among 2297 patients undergoing surgery for uterine fibroids
